# Pedal Claw Curvature in Birds, Lizards and Mesozoic Dinosaurs – Complicated Categories and Compensating for Mass-Specific and Phylogenetic Control

**DOI:** 10.1371/journal.pone.0050555

**Published:** 2012-12-05

**Authors:** Aleksandra V. Birn-Jeffery, Charlotte E. Miller, Darren Naish, Emily J. Rayfield, David W. E. Hone

**Affiliations:** 1 Structure and Motion Lab, Royal Veterinary College, Hatfield, Hertfordshire, United Kingdom; 2 School of Earth Sciences, University of Bristol, Wills Memorial Building, Bristol, United Kingdom; 3 Evolutionary Anthropology, Duke University, Durham, North Carolina, United States of America; 4 Ocean and Earth Science, National Oceanography Centre, University of Southampton, Southampton, United Kingdom; 5 School of Biological and Chemical Sciences, Queen Mary University of London, London, United Kingdom; 6 School of Biology and Environmental Science, University College Dublin, Dublin, Ireland; University of Pennsylvania, United States of America

## Abstract

Pedal claw geometry can be used to predict behaviour in extant tetrapods and has frequently been used as an indicator of lifestyle and ecology in Mesozoic birds and other fossil reptiles, sometimes without acknowledgement of the caveat that data from other aspects of morphology and proportions also need to be considered. Variation in styles of measurement (both inner and outer claw curvature angles) has made it difficult to compare results across studies, as have over-simplified ecological categories. We sought to increase sample size in a new analysis devised to test claw geometry against ecological niche. We found that taxa from different behavioural categories overlapped extensively in claw geometry. Whilst most taxa plotted as predicted, some fossil taxa were recovered in unexpected positions. Inner and outer claw curvatures were statistically correlated, and both correlated with relative claw robusticity (mid-point claw height). We corrected for mass and phylogeny, as both likely influence claw morphology. We conclude that there is no strong mass-specific effect on claw curvature; furthermore, correlations between claw geometry and behaviour are consistent across disparate clades. By using independent contrasts to correct for phylogeny, we found little significant relationship between claw geometry and behaviour. ‘Ground-dweller’ claws are less curved and relatively dorsoventrally deep relative to those of other behavioural categories; beyond this it is difficult to assign an explicit category to a claw based purely on geometry.

## Introduction

Claws perform a variety of functions in tetrapods and typically represent the first and last contact an animal has with the substrate during locomotion. In extant birds, lizards and other tetrapods, claw morphology has been related to behaviour, ecology and prey capture method [1], [2], [3], [4], [5], [6], [7], [8]. In a diverse literature, mostly focused on Mesozoic birds and bird-like theropod dinosaurs, workers have aimed to relate claw morphology with behaviour (e.g. [1], [2], [9], [10], [11], [12]).

The tetrapod claw is a composite structure composed of an inner bony core (the ungual bone) sheathed by keratin. Keratin is a highly specialised epidermal derivative [13], with its functionality in claws being little known. Previous work on the material properties of keratin have focused on human tissues as well as beaks, horns and hooves [14], [15], [16]. Birds and reptiles both possess β-keratin, a form of keratin known to be harder than the alpha keratin found in humans and other mammals [17], [18], [19]. The keratin sheath of a claw is constantly abraded, especially at the tip; this is thicker to counter excessive abrasion and is continuously renewed internally [20], [21], [22]. The keratin sheath sometimes extends well beyond the length of the ungual, so may increase both the length and curvature of the claw in life [11].

It has long been understood that claw form may be informative with regard to an animal’s lifestyle, and several authors have made qualitative generalisations linking claw form with behaviour and/or lifestyle. Gross observations of claw shape in extant birds and mammals show that terrestrial taxa possess weakly curved claws, perchers possess conical, strongly curved claws, predatory birds possess tapered claws with marked curvature, and scansorial (trunk-climbing or clinging) birds and mammals possess laterally compressed claws with needle-like points (e.g. [1], [23], [24], [25]). However, subjectivity is inherent in determining whether a claw is ‘weakly’ or ‘strongly’ curved.

Determining the claw curvature type in a fossil taxon may be controversial and this has certainly been the case in the Jurassic taxon *Archaeopteryx*. Some authors have supported scansoriality or arboreality in *Archaeopteryx* [1], [2], [26], [27], [28], [29] while others have argued that its pedal claw form is inconsistent with a climbing lifestyle and better indicates terrestriality [9], [12], [23], [30]. Analysis of the claw types present in extinct taxa has not been confined to *Archaeopteryx*; claw geometry analysis has been applied to many Mesozoic theropods [11], [30], [31], [32] and to non-dinosaurian fossil reptiles [10].

In an effort to quantify claw architecture, authors have used claw curvature data, from extant animals, to hypothesise on relationships between claw curvature, broader morphology and lifestyle in extinct species. Feduccia [2] was the first to describe this relationship in extant birds. A measure of inner claw curvature (i.e. curvature along the ventral edge) was plotted within three broad behavioural categories. Higher curvature indicated greater specialisation for arboreality; however, birds with claws specialised for specific tasks (e.g. predation, digging) were not included in the analysis. Furthermore, there was substantial overlap of data between the categories of habitat use, limiting their explanatory powers.

Several later studies also examined claw geometry in birds as well as in lizards; all have claimed some degree of correlation between claw form and behaviour [4], [6], [7]. However, these studies have employed different methodologies (even within analyses of the same curvature) in measuring claw geometries: inner claw curvature [2], [3], [7] and outer claw curvature [4], [5], [6] have both been used, making it difficult to directly compare their results.

Another issue when comparing studies is the relatively small amount of overlap in the list of taxa analysed. Feduccia [2] analysed a variety of ‘ground-dwellers’, ‘perchers’ and ‘climbers’ but avoided species with ‘unusual adaptations,’ such as predatory birds. Glen and Bennett [6] argued that Feduccia’s behavioural categories oversimplified the continuum that exists between committed ‘ground-dwellers’ and truly arboreal species within Aves and instead categorised species based on the amount of time spent foraging in terrestrial or arboreal habitats. They then limited their study to Columbiformes and Cuculiformes as these were sufficiently diverse in behaviour and body mass, and lacked foot specialisation that might confound the analysis. Other geometry studies have sampled phylogenetically diverse arrays of bird [4] and lizard species [3], [7]. All of these studies reported some relationship between claw curvature and lifestyle, with claw curvature increasing with time spent climbing.

Whilst all claw curvature studies found some relationship between curvature and behaviour, they often disagreed on the finer points. Studies on lizard claws have reported significant increases in clinging performance with increases in claw curvature, toe width and/or claw height [3], [33]. This corresponds well with the findings of Feduccia [2] and Glen and Bennett [6]: increased curvature indicates greater arboreality. Conversely, Pike and Maitland [4] disagreed, noting that – whilst separations between certain behavioural categories could be identified (i.e. ‘climbers’ versus ‘ground-dwellers’) – large overlaps in claw curvature meant that distinct separations between behavioural categories were not apparent.

Given that claw geometry studies cannot necessarily determine function from claw shape, authors have used other metrics in efforts to demonstrate a relationship. Members of Falconidae and Accipitridae can be distinguished on grip force data, for example, with grip force capacity generally being higher in Acciptridae, as is consistent with prey capture methods [34], [35]. Extant raptors can also be separated on the basis of interdigital variation [8]; indeed, Hopson [36] described how phalangeal proportions could be used to differentiate behaviours across birds. Several lines of evidence therefore suggest that similar claw geometries may not necessarily indicate analogous claw function.

Indeed, singular morphological features may not always provide a reliable guide to behaviour, since different structures can perform similar functions [37]. Furthermore, morphology often represents a compromise caused by the need for multi-functionality; like other structures, claws perform diverse functions in an animal’s life and interact with different surfaces. Inferences about extinct species therefore need to be made with the appropriate caveats in mind.

Data on a growing list of taxa has improved our understanding of the patterns that relate claw shape to behaviour and ecology, but further research is still required in order to better establish relationships in extant animals, and we have some way to go before we have a chance of accurately inferring claw function in extinct taxa.

In this study we aimed to test whether claw morphology can be used to predict arboreal or terrestrial habits in Mesozoic birds and other coelurosaurian theropods. We firstly analyse extant bird and lizard claws to determine the strength of the relationship between behaviour and morphology, and to test if the chosen methodology will affect this relationship. We also test whether phylogenetic control has a strong impact on the behavioural signal. We then compare these results to data gleaned from fossil taxa.

## Methods

We examined claw morphology in a diverse dataset of extant species belonging to Aves and Squamata, thus sampling members of an extended extant phylogenetic bracket for Mesozoic dinosaurs [38]. Crocodylians were not used as all living species are semi-aquatic and thus may confound the results. In the absence of a more suitable extant out-group to Aves, we used the behaviourally diverse squamates to facilitate an understanding of the link between claw shape and behaviour. Within both our bird and squamate datasets, we deliberately selected a phylogenetically and behaviourally diverse range of species.

All specimens were photographed in lateral view (see Table S1 for specimen details); specimens used were from public museum collections and were photographed on site.

Up to six adult specimens (details in Table S1) were measured for each sampled species; ontogenetic changes in claw forms are unknown and many taxa show behavioural changes during ontogeny that could conceivably affect claw geometry. Following the rationale of previous claw studies [2], [4], [6], pedal digit III was used for the main analysis since it is longest in birds and in non-avialan Mesozoic theropods. Digit III therefore contacts the substrate first and last during terrestrial locomotion in these taxa. Unguals for digits I, II and IV were also photographed and measured in order to ensure that the results presented here are not an artefact of using digit III alone. In the text, unless specifically attributed to digits I, II or IV, our results refer to digit III analyses. All extant animal claws used in this study were in possession of their keratinous sheath (except in the case of three squamate specimens which were used due to lack of other squamates – see Table S1 and S8 for details).

We supplemented our database of museum specimens with data from Pike and Maitland [4] being added to our own. Data from fossil specimens were selected from the literature, or collected firsthand (see Table S2). The majority of fossil specimens lacked both the claw’s keratinous sheath and any impression of it; in these cases the ungual alone was used. Where a keratinous sheath was present, both the ungual and the keratinous sheath were measured.

**Figure 1:**
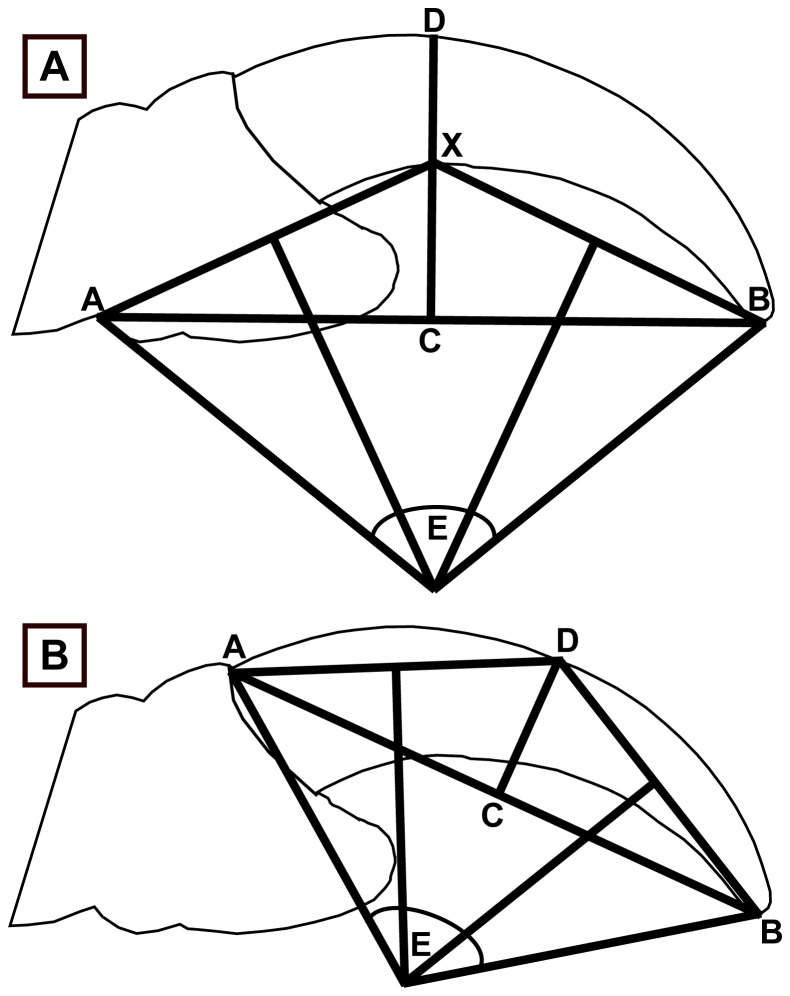
Representation of curvature measurements. (A) Inner claw curvature after Feduccia (1993). Relative midpoint height is a ratio of lengths CX and CD. Curvature is the angle E in both methods. Point A is the proximal ungual and line AB is drawn to finish at point B, the claw tip. Line CD is drawn perpendicular to line AB and X is the point where CD crosses the inner curvature of the claw, and joined together with points A and B. Perpendicular lines are drawn between AX and BX. Where these two lines meet lines A and B are drawn to create curvature E. (B) Outer claw curvature after Pike & Maitland (2004), only curvature E was only measured here as in highly curved claws, the line ACB dissected the claw making the measurement of midpoint height void. Point A was selected as the outermost point of the claw before the scale covering and B was the claw tip. Line CD was drawn perpendicular to AB and again perpendicular lines were drawn from AD and BD to create curvature E.

Geometric measurements were recorded using Feduccia’s [2] methods for inner claw curvature (Fig. 1A) and Pike and Maitland’s [4] method for outer claw curvature (Fig. 1B) with the addition of relative claw robusticity: the dorsoventral height of the claw at its midpoint (ratio of lines CX and CD in Fig. 1A). A total of 832 specimens of 331 species were measured: these were placed into four behavioural categories depending on their dominant form of locomotion taken from descriptive accounts of their lifestyle [39], [40], [41], [42], [43], [44], [45], [46], [47], [48], [49], [50], [51]. These were: ‘predatory’ (bird: inner  = 146, outer  = 178; lizard: none), ‘climbers’ (bird: inner  = 26, outer  = 40; lizard: inner  = 5, outer  = 4), ‘perchers’ (bird: inner  = 172, outer  = 157; lizard: none) and ‘ground-dwellers’ (bird: inner  = 323, outer  = 319; lizard: inner  = 38, outer  = 34). Photos were scaled in ImageJ 1.38× software [52] where the selected variables were measured. Bird body mass data were taken from Dunning [53] and averages taken if both male and female data were available. Squamate masses were taken from the literature (Table S3). Masses of extant taxa were examined to determine if a mass-specific effect could be observed in the data. Masses for extinct taxa were not estimated.

Graphical summaries and statistical analyses were performed in SPSS 16.0 (for Windows, Release 17.0.1 2008, Chicago, SPSS Inc.), PAST [54] and R [55]. A generalised least squares regression (GLS) model was performed on body mass and curvature data. A weighting was added to the data to account for the different variances within behaviour groups (heteroscedasticity); the model rescales these variances depending on behaviour group. Regression analyses were performed per behavioural category on body mass and curvature. Finally, due to differing variances, a non-parametric median test was performed in order to find significant differences in the median curvatures between the behavioural groups. The statistically significant p-value was kept at the standard 0.05.

### Independent Contrasts

Independent contrasts provide a powerful tool in assessing the effects of phylogeny on continuous traits. Independent contrasts through Felsenstein’s methods [56] do not require accurate knowledge of branch lengths or the assumption of gradual character evolution [57], though it has been noted that species selection and branch length can play a significant role in the accuracy of an analysis, since minor changes can give varying Type I error rates [58]. A single phylogeny encompassing a truly representative assemblage of bird species has not yet been produced: we used three different phylogenies in an effort to analyse the effect of behavioural categories on claw morphology. Inner claw curvature was used as it allowed us to include relative mid-point height measurements in the analysis and observe its effects on behavioural category. We used Livezey and Zusi’s [59] comprehensive phylogenetic analysis of morphological characters, and Ericson *et al.*’s [60] molecular phylogeny, as both incorporate a large, phylogenetically representative list of bird taxa. A more recent large-scale molecular phylogeny of extant birds has since been published [61] but this lacks the generic level details required for our analysis. Lastly, a combination matrix was created using the Livezey and Zusi [59] phylogeny in order to establish relationships amongst bird clades; separate phylogenies were then used to determine the relationships within each clade (Table S4). Our aim here was to try and encompass as many of the measured species as possible. The phylogenetic position of congeneric species was used to indicate the position of species not contained within the phylogenetic analysis (Table S5). Once phylogenies were created, each species was coded within the program CAIC [62] and independent contrasts were calculated.

## Results

### Geometric Measurements

We used Spearman’s correlation coefficient test (*r_s_*) to determine whether or not inner and outer digit III claw curvatures were correlated. Correlation was found to be significant across extant taxa (*r_s_* = 0.510; p<0.005), and similarly in avian-only data (*r_s_* = 0.516; p<0.005). Thus, in all instances, both inner and outer curvature of the ungual of digit III increased with increasingly arboreal life habits. The squamate data for digit III were not significantly correlated (*r_s_* = 0.184; p<0.134), but this may be due lack of power in non-parametric tests with small data sets (the squamate data included only 43 specimens).

#### Relationships between digits

We compared data across the digits of extant birds and lizards, the aim being to observe whether digit III values were different from those of other digits. A preliminary test (median test) showed that inner curvature (p = 0.003), mid-point claw height (p<0.005) and outer curvature (p<0.005) were all significantly different across the respective digits (see Table S8 for dataset). The dataset type (all extant animals or bird only) did not affect the result. We further analysed whether this affect occurred when taxa were split into behavioural categories. We observed if the median of curvatures and mid-point claw height remained constant across behavioural categories. Inner curvature only showed a significant difference in ‘ground-dweller’ and ‘predatory’ categories (p = 0.047; p = 0.024); though these were small as adjusted pairwise comparisons failed to show where the disparity occurred. Mid-point claw height was significantly different in ‘percher’ and ‘predatory’ categories (p<0.005; p = 0.007); in both cases, digit I was significantly thicker relative to all other digits. Outer curvature showed significant differences within the behavioural categories: ‘ground-dweller’ (p = 0.027), ‘percher’ (p<0.005) and ‘predatory’ (p<0.005). Unlike the inner curvature, in the outer curvature digit I was not consistently significantly different to other digits. In all categories, digit III had a lower curvature. In ‘ground-dwellers’, curvature in digit III was significantly lower than in digit IV, in ‘perchers’, curvature was lower in digit III than digits I, II and IV and, finally, curvature in ‘predatory’ taxa was lower in digit III than in digits I and II.

#### Relationship to body mass

**Figure 2:**
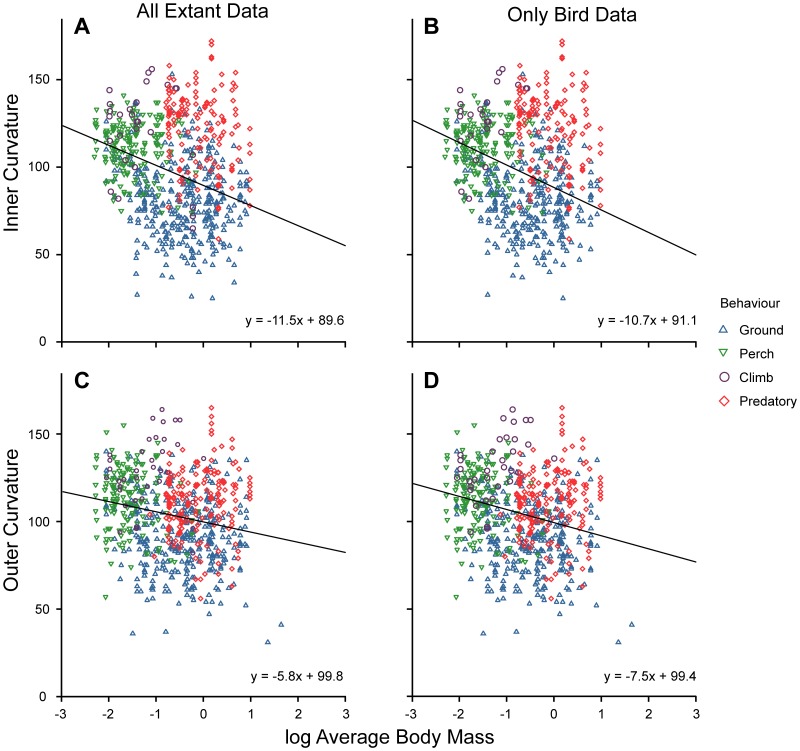
Regression plots for digit III curvature of combined behavioural categories against log body mass. (A) Inner curvature for all extant data (birds+squamates); (B) Inner curvature for all extant bird data only; (C) Outer curvature for all extant data; (D) Outer curvature extant birds only.

Body mass is related to both inner and outer digit III claw curvature in birds (Fig. 2). When all behavioural data are combined, a negative relationship is discovered and the removal of the phylogenetically distant lizards does not alter the signal. The confidence intervals of the bird and combined extant (bird+lizard) datasets overlap (Table 1). In all instances there is a large amount of scatter when the data are not split into separate behavioural categories. When we excluded lizard data, the AIC (Akaike’s Information Criterion: a measure of the fit of the model using R) decreased in both inner and outer curvature: the change was small, however. This confirms that the addition of lizard data does not obscure the relationship between curvature and body mass. Unlike curvature, claw mid-point height is positively related to body mass (p<0.005; y = 0.04 (±0.01) x+0.48 (±0.01) – data not shown). Again, the removal of the lizard dataset slightly shifts the regression equation; the confidence intervals overlap one another while the AIC is only slightly smaller. For these reasons, individual regression analyses of curvatures on body mass were performed on each behavioural category for the full dataset (lizards were not excluded).

**Table 1:** Results from a generalised least squares regression on curvature against body mass.

Curvature	Data set	Slope (±CI)	Intercept (±CI)	p-value
**Inner**	**All**	−11.5 (2.1)	89.6 (2.6)	<0.001*
	**Bird Only**	−10.7 (2.2)	91.1 (2.7)	<0.001*
**Outer**	**All**	−5.8 (1.9)	99.8 (2.1)	<0.001*
	**Bird Only**	−7.5 (2.0)	99.4 (2.3)	<0.001*

* denotes a significant p-value (<0.05).

When the inner curvature data are split into behavioural categories, only the ‘ground-dweller’ and ‘predatory’ categories showed a significant relationship with body mass (p = 0.004; p = 0.015, respectively), with both being negatively related. In outer curvature only the ‘ground-dweller’ category was negatively related to body mass (p = 0.005). However, in all categories the amount of data explained (R^2^) by these linear best fit lines was low, with a maximum of 0.03 in the inner curvature ‘predatory’ category. This indicates that other factors are influencing the relationship between these two variables, as shown by the spread of the data (Fig. 2). ‘Predatory’, ‘climber’ and ‘ground-dweller’ categories were significantly related to body mass by claw mid-point height (p<0.005; p = 0.040; p<0.005 respectively), and although R^2^ values are higher than for the curvature relationships, the fit is still low (maximum R^2^ of 0.11 found in the ‘climber’ category).

### Extant Data Behavioural Categories

**Figure 3:**
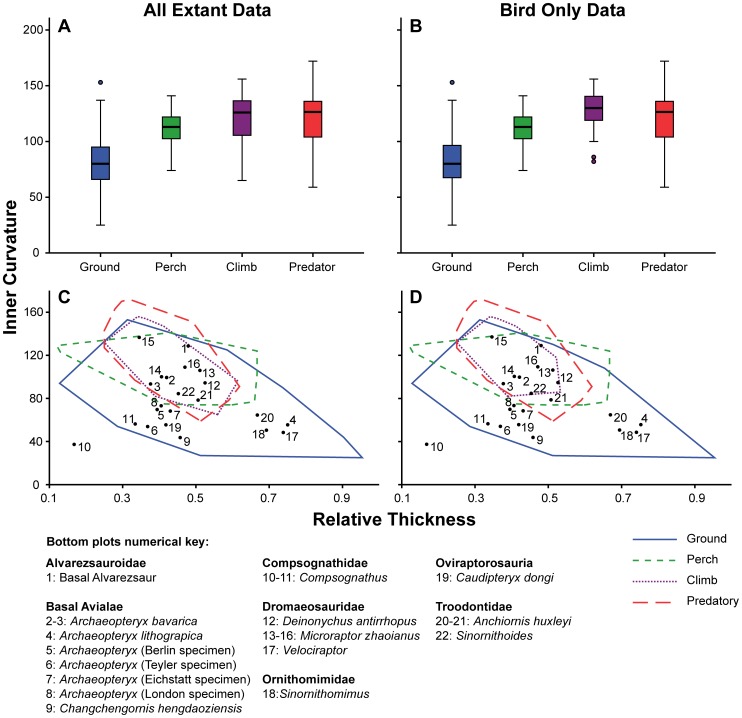
Inner claw curvature, digit III, for all extant taxa and Mesozoic theropods. Box plots for inner claw curvature distinguished by behavioural category for (A) all extant taxa, (B) extant birds only. Shaded boxes depict the interquartile range (IQR) with median marked as the horizontal line. The whiskers indicate the distance between the IQR and points up to 1.5 distances from the IQR. Outliers are represented as circles and are between 1.5 and 3 distances from the IQR. Inner claw curvature plotted against relative midpoint height, overlain with Mesozoic theropod data for (C) all extant taxa, (D) extant birds only.

**Figure 4:**
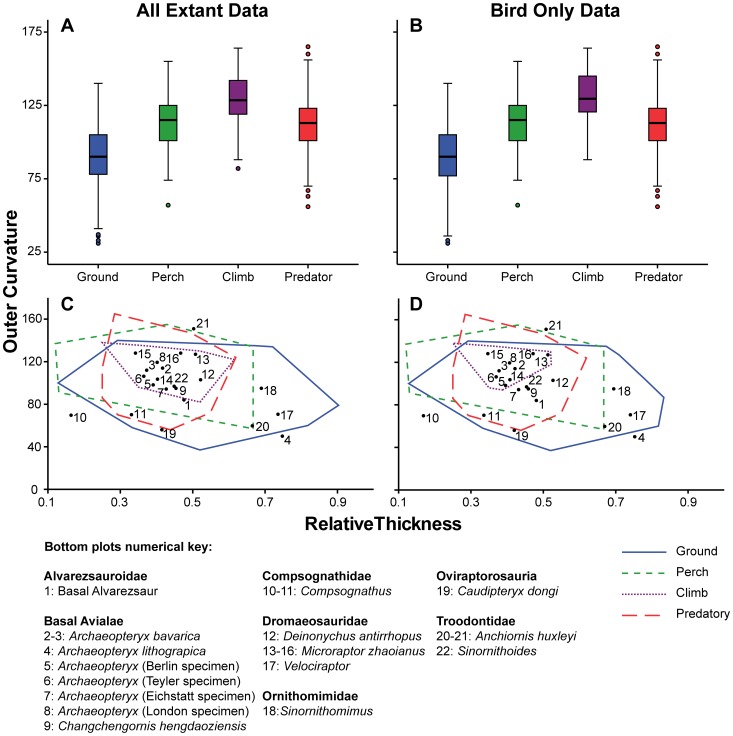
Outer claw curvature, digit III, for all extant taxa and Mesozoic theropods. Box plots for outer claw curvature distinguished by behavioural category for (A) all extant taxa, (B) extant birds only. Shaded boxes depict the interquartile range (IQR) with median marked as the horizontal line. The whiskers indicate the distance between the IQR and points up to 1.5 distances from the IQR. Outliers are represented as circles and are between 1.5 and 3 distances from the IQR. Outer claw curvature plotted against relative midpoint height, overlain with Mesozoic theropod data for (C) all extant taxa, (D) extant birds only.

Claws in the ‘ground-dweller’ category encompassed the largest range in both inner (Fig. 3) and outer (Fig. 4) digit III curvature measurements. Ranges of data were smaller in all other categories, but these categories did include a smaller number of specimens. Median values of outer curvature were significantly greater than those of inner curvature in all but the ‘ground-dweller’ category.

The median of inner curvature differed significantly between all behavioural categories (Tables 2 and 3) except the ‘predatory’ and ‘climber’ categories in both the combined and avian-only datasets. This is not surprising as the box plot shows similar ranges, medians and interquartile ranges (IQR) for the two categories (Fig. 3A, B). There is a consistent outlier in inner curvature among the ‘ground-dweller’ data: *Ptilonorhynchus violaceus* (Satin bowerbird). The curvature is higher than 1.5 IQRs (from median) in the ‘ground-dweller’ category. In the bird-only dataset, two further outliers are apparent in the ‘climber’ category, showing lower curvature than the rest of the data: *Zosterops japonicus* (Japanese white-eye) and *Hirundo rustica* (Barn swallow). The latter taxon was difficult to assign to a behavioural category, but was placed within the climbers due to the tendency of it and other hirundine passerines to cling vertically to mud nests and vertical breeding cliffs [63]. Though medians statistically differed, there is a large overlap in data between all categories, with data from the ‘ground-dweller’ category overlapping the others. It appears that the inner curvature median increases in digit III as the animal’s lifestyle becomes more dependent on arboreality or on predation. A comparison of relative claw mid-point height to inner curvature was performed and convex hulls were used to display the smallest polygon encompassing all data points in an extant behavioural category. This shows in greater detail that the morphospace occupied by the categories involves extensive overlap between climbers and predators and notable overlap of these two categories with perching taxa and some ground-dweller birds (Figs. 3C, D). Only data from the ‘ground-dweller’ category extends beyond a range of 0.7 in claw mid-point height.

**Table 2:** Results for the non-parametric median test and the pairwise comparisons.

	Curvature	Test Statistic (d.f.)	p-value
**All extant data**	**Inner**	273.5 (3, 711)	<0.001*
	**Outer**	150.7 (3, 732)	<0.001*
**Bird only data**	**Inner**	246.3 (3, 668)	<0.001*
	**Outer**	149.6 (3, 694)	<0.001*

* denotes a significant p-value (<0.05).

**Table 3:** Pairwise comparisons within each behavioural category.

	All Extant Data				Bird Only Data			
Inner Curvature	Ground	Perch	Climb	Predatory	Ground	Perch	Climb	Predatory
**Ground**	–	–	–	–	–	–	–	–
**Perch**	<0.001*	–	–	–	<0.001*	–	–	–
**Climb**	<0.001*	0.042	–	–	<0.001*	0.001*	–	–
**Predatory**	<0.001*	<0.001*	1	–	<0.001*	<0.001*	1	
**Outer Curvature**				–				
**Ground**	–	–	–	–	–	–	–	–
**Perch**	<0.001*	–	–	–	<0.001*	–	–	–
**Climb**	<0.001*	<0.001*	–	–	<0.001*	<0.001*	–	–
**Predatory**	<0.001*	1	<0.001*	–	<0.001*	1	<0.001*	–

* denotes a significant p-value (<0.05).

The outer curvature medians also differed significantly from one another (Table 3): however, in this case, the only non-significant pair-wise comparison was between the ‘predatory’ and ‘percher’ categories. As with inner curvature, removing the lizard data from this analysis had no effect on results. Unlike inner curvature, the ‘climber’ category has a much higher outer claw curvature, with ‘predatory’ and ‘percher’ categories having very similar ranges (Fig. 4). As is the case with inner curvature, the ‘ground-dweller’ category had the largest range and the lowest median value. The outer curvature data had more outliers than the inner curvature data, though there were still no extreme points (over 3 times from the IQR). In the combined extant dataset the outliers for the ‘ground-dweller’ category were *Himantopus himantopus* (Black-winged stilt), *Otis tarda* (Great bustard), *Eremophila alpestris* (Shore lark), and *Rhea americana* (Greater rhea), all of which had lower curvature than expected. In the ‘climber’ category, *Chlamydosaurus kingii* (Frill-necked lizard) possessed lower curvature than expected. Finally, in the ‘predatory’ category, *Buteo buteo* (Common buzzard), *Sagittarius serpentarius* (Secretary bird) and *Tyto tenebricosa* (Greater sooty owl) all exhibited lower curvature than expected, whilst *Pandion haliaetus* (Osprey) had higher curvature. These outliers changed slightly when the squamate data were removed; alongside the frill-necked lizard outlier, the black-winged stilt and the shore lark are no longer outliers.

### Comparison with Mesozoic Theropods

Data for Mesozoic maniraptorans and other coelurosaurs was plotted on top of extant data convex hulls, showing that extant and extinct data occupied similar areas in plots of inner or outer curvature against relative claw mid-point height for digit III claws (Figs. 3 C, D & 4 C & D).

For the inner curvature data there is an even split of Mesozoic taxa between the ‘ground-dweller’ convex hull and the other categories of ‘climber’, ‘percher’ and ‘predatory’. In the total dataset and bird-only one, there was only one outlier: one of the two data points for the compsognathid *Compsognathus* (point 10). The other *Compsognathus* point (point 11, the Solnhofen specimen described by Ostrom [64]) lies just within the ‘ground-dweller’ convex hull but both points exhibit low relative mid-point height combined with low claw curvature. All dromaeosaurids bar *Velociraptor* are found within the ‘climber’ – ‘predatory’ – ‘percher’ – ‘ground-dweller’ shared space. These Mesozoic coelurosaurs show medium claw mid-point height combined with high curvatures. Two of the three troodontid points lie within the ‘climber’ – ‘predatory’ – ‘percher’ – ‘ground-dweller’ shared space. The other point (20: *Anchiornis huxleyi* [65]) lies in the exclusive ‘ground-dweller’ region, due to its greater relative mid-point height. Among other Mesozoic coelurosaurs, only two points lie in the more arboreal habitats; the majority lie only within the ‘ground-dweller’ convex hull. The spread of *Archaeopteryx* claws is the largest, showing up in low-medium curvatures and also low to high relative mid-point height. Interestingly, the *A. bavarica* specimens lie within the shared space, whilst *A. lithographica* claws occupy the exclusive ‘ground-dweller’ space within our analysis.

In digit III outer curvature (Figs. 4C, D), a large portion of the Mesozoic coelurosaurian points fall within the ‘percher’ – ‘climber’ – ‘predatory’ – ‘ground-dweller’ convex hulls. Three outliers exist, outside of the ground convex hull: one specimen of each *Compsognathus*, *Anchiornis* and *Archaeopteryx*. Here, the Mesozoic coelurosaurs *Sinornithomimus, Caudipteryx* and *Velociraptor* lie only within the ‘ground-dweller’ category. *Anchiornis* (20) sits just within the shared ‘ground-dweller’ – ‘percher’ space. Note that there is a large discrepancy between the two *Anchiornis* points since both exhibit different claw curvatures. Unlike inner curvature, here all the *Archaeopteryx* and *Changchengornis* points fit remarkably well into the cluster of extant ‘climber’ category in all extant data. Only the Eichstätt specimen of *Archaeopteryx* falls outside this category in the birds-only dataset (point 7, Fig. 4D). *Compsognathus* retains a remarkably similar position relative to the convex hulls, as it did in inner curvature.

#### Independent contrasts

The number of species used in each independent contrasts analysis depended on the species in the phylogenetic study and species in this current study, which did not necessarily match. Use of Livezey and Zusi’s [59] bird phylogeny produced no significant relationship between inner claw curvature and behaviour for digit III (F_9_ = 2.84; p = 0.130; r^2^ 0.262). Sixty-two species were used for this analysis with no outliers being found in the analysis (see Table S6). Fewer species were used (n = 36) in the analysis using the Ericson *et al.* [60] phylogeny (see Table S7). This analysis again showed no outliers, but had even lower amounts of explained data and non-significant results (F_7_ = 0.89; p = 0.385; r^2^ = 0.128). The ‘combination method’ phylogeny gave the highest number of species for analysis (n = 164); these were distributed across 16 bird orders. A marginally significant result was found (F_26_ = 4.22; p = 0.051; r^2^ = 0.144). However, 11.54% of the data points used in this analysis were recovered as outliers, indicating that a large portion lay outside the average dataset.

## Discussion

Many previous studies have attempted to link claw geometry with habitat use and hence lifestyles, mostly in an effort to better understand the ecology of Mesozoic birds and their close relatives within Maniraptora [2], [6], [11], [32]. The next step would be to expand existing datasets with the addition of new taxa and more individuals of sampled taxa. The dataset employed in our study is phylogenetically broader and more comprehensive than that of previous studies as we aimed to see whether claw geometry varied independently of phylogenetic control.

Previous studies have not compared the effect of adding a phylogenetically distant group to comparisons of claw geometry; our analysis shows that adding squamates to an analysis of birds does not appear to change the results (Figs. 2, 3 and 4). This allows us to be more confident in asserting that trends in claw morphology occur across tetrapods, and thus allows us to plot Mesozoic coelurosaur data over data on extant taxa. However, when the data were made independent by removing the effects of phylogenetic relatedness (using independent contrasts), no correlation was recovered between behavioural category and claw curvature for digit III. This result differs from previous studies on lizard claw morphology [3], [33] and bird claw morphology [6] where phylogeny was accounted for. The combination phylogeny for birds showed marginal significance between behaviour and curvature, but this result was diminished by the lack of explained data.

Our comparisons between claws of different digits suggest that results would differ depending on which claw was used, though note that the differences were not necessarily significant once *post hoc* comparisons were made (comparisons suggestive of small disparities between the respective digits). This effect appears to be intensified in outer claw curvature, where only the climbing category showed no significant differences between digits. One possible explanation for this result is that, even though the inner claw contacts the substrate, it is the outer curvature of the claw that is more closely linked to strength and hence linked to the degree of stress or strain that the claw can withstand. Another possible explanation may come from the fact that digits differ in length, with some extending further from the tarsometatarsal-phalangeal joint than others: a consequence being that they have different functions. This will lead to different claw forms on different digits. Inner claw curvature, though, is not as affected by different digits as outer claw curvature.

Curvature of the digit III claw is very weakly correlated to body mass, with a general trend of decreasing curvature with increasing mass. The correlation is poorer than that reported in previous studies [4], [66], with behavioural categories showing opposite trends to the results found here. The claw mid-point height, however, correlates well with mass, similar to the result reported by Pike and Maitland [4].

It is important to note that substantial overlaps between behavioural categories have previously been found [2], [4] and furthermore that behavioural categorisation is fundamentally difficult: most animals do not demonstrate a single behaviour but usually several different ones to varying degrees (i.e. claws can be multi-functional and even only occasional use may be strongly selected for if sufficiently important to the animal’s fitness). For example, predatory birds also perch, and birds with strong perching or climbing adaptations can still spend a considerable time foraging on the ground. Our results agree with previous work [4] in finding it difficult to take a specific claw and place it into a restricted behavioural category (Figs. 3–4). These results suggest that multi-functionality or compromise in claw shape is common, perhaps ubiquitous, and that a few simple metrics of claw shape are unlikely to give a truly clear picture of the ecology or behaviour of the species concerned. We can state with some confidence that the claws of ‘ground-dweller’ taxa can be separated from those present in other behavioural categories based on the data collected here for the ungual on digit III. ‘Ground-dwelling’ claws have higher relative claw mid-point height and lower curvature, but separating out the other categories is difficult.

Pike and Maitland [4] suggested that ‘perchers’ are behaviourally generalised with regards to their non-specific claw shape, but their study only incorporated ‘perchers’ from among Passeriformes. ‘Perchers’, however, are found in other major avian clades and representatives of the species concerned have been included in this study. In contrast to Pike and Maitland [4] we find that ‘ground-dwellers’ are, behaviourally, the most generalised group with the largest spread of curvature and mid-point height values in digit III claws (Figs. 3 & 4). ‘Ground-dwellers’ are also more broadly distributed phylogenetically. ‘Perchers’ on the other hand appear to have very similar curved claws and it appears logical that perching and climbing behaviour would require greater specialisation with regards to curvature. We hypothesise that ‘ground-dweller’ claws require fewer morphological and biomechanical constraints in order to function effectively.

**Figure 5:**
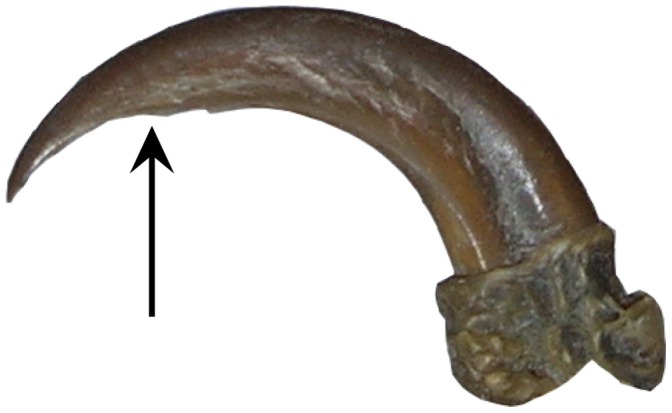
Pedal claw of *Campephilus melanoleucus* (Crimson-crested woodpecker). Arrow indicates the constriction.

Claws suited for climbing appear to often possess a constricted region in the proximal part of their ventral curvature (Fig. 5). Constrictions are suggested to be climbing aids and are typically present in treecreepers and woodpeckers [4]. However, they were not present in all climber-type claws incorporated in our study of museum specimens, being absent in *Loriculus beryllinus* (Psittaciformes), *Margarornis squamiger* (Passeriformes) and *Picus viridis* (Piciformes). The absence of constricted claws could conceivably be due to lack of normal wear stemming from time spent in captivity, or it may be that the specimens concerned belong to species that truly lack them. However, their absence in some taxa suggests that, while they may be advantageous for climbing, they are not essential. Further research is required in order to investigate how much of an effect these constrictions have on climbing (and possibly perching) birds and whether the constriction is present only in the sheath or is present on the ungual. One further result of note is that a higher than expected curvature is seen in the osprey *Pandion haliaetus*, which may be due to its specialisation for gripping fish. Though other piscivorous raptor species were not measured in this study (nor were piscivorous owls or bats) this result agrees with findings that *Pandion* has enlarged, highly recurved claws on each pedal digit consistent with its prey capture and restraint strategy [8].

In addition to extant taxa, we sampled digit III claws for 22 specimens from 11 genera of representative Mesozoic birds and other coelurosaurian theropods. Our data suggests that many Mesozoic coelurosaurs were terrestrial (Fig. 3). Only three specimens fall distinctly outside of the ‘ground-dweller’ zone, and these all fall outside of all extant behavioural categories (Fig. 4). Among fossil coelurosaurs, various Mesozoic maniraptorans possess features apparently consistent with at least some tree-climbing ability [26], [31], [67], [68]. In outer claw curvature, both dromaeosaurids (*Deinonychus*, *Microraptor*) and Mesozoic maniraptorans conventionally identified as birds (*Archaeopteryx*) clustered within an area encapsulated by the extant ‘climber’ category.

However, the range of results obtained for the various Mesozoic taxa is striking. The various *Archaeopteryx* specimens are well scattered; *A. lithographica* is largely recovered within the ‘ground-dweller’ hull while *A. bavarica* falls within the space shared by all behavioural categories. Collectively, these specimens possess both the highest and one of the lowest values for claw mid-point height (Fig. 3), meaning that quantifying their behaviour is difficult based on digit III. Each specimen of *Microraptor zhaoianus* lies within the ‘climber’ hull suggesting that this maniraptoran at least had claws suitable for climbing. This finding differs notably from that reported by Dececchi and Larsson [12] where both *Archaeopteryx* and *Microraptor* did not fall within the same regions of morphospace as scansorial mammals, lizards or birds based on a number of anatomical features and ratios. This large discrepancy may result from the inclusion of mammals in the study [12]: a much more distant group in phylogenetic terms, the members of which have evolved different adaptations to an arboreal lifestyle. Surprisingly, *Velociraptor* lies well within the ‘ground-dweller’ category and not within the ‘predatory’ area, despite its carnivorous lifestyle. Manning et al. [32] concluded that “the feet and hands of dromaeosaurs functioned both for locomotion (walking, running, and climbing) and as prey capture/grappling devices”, though our data for digit III supports only a terrestrial interpretation. *Deinonychus*, however, does fall within the predatory category, suggesting that its foot may have been used in prey immobilisation behaviour analogous to modern Accipitridae [66], [69].

While our dataset is limited, and the hulls overlap extensively, it is still plausible based on our data for digit III claws that some Mesozoic maniraptorans were tree-climbers. Based on outer curvature data, both *A. bavarica* and *Changchengornis* appear to be ‘climbers’, although inner claw curvature places *A. lithographica* in the ‘ground-dweller’ category. *Anchiornis* and *Microraptor* have the potential to be considered ‘perchers,’ and the latter also has characteristics consistent with some climbing ability. Interestingly, *Anchiornis* lies in similar places in both inner and outer curvatures but in some instances of outer curvature lies outside of the extant data used here. Our data on Mesozoic maniraptorans cannot be interpreted as providing strong evidence for any level of arboreality, due to the lack of clear relationship between geometry and behaviour, and the dependency on evolutionary history.

We included several species known to use wing-assisted incline running (WAIR): Chukar partridge (*Alectoris chukar*), Rock dove (*Columba livia*), Black-billed magpie (*Pica pica*) and Brush turkey (*Alectura lathami*) [70]. These species all belong to either the ‘ground-dweller’ or ‘percher’ category based on claw shape. In both inner curvature and relative claw mid-point height they encompass a large range (77–129° and 0.38–0.66, respectively), but their range of outer curvature is relatively small (66–80), possibly due to smaller sample size. Due to the large inner curvature range we can assume that WAIR capability does not require a specialised claw form, though further investigations are required to determine whether the trend continues in outer curvature. This may therefore suggest that some troodontids, dromaeosaurids, and possibly *Archaeopteryx*, would have been capable of WAIR.

Collectively, the results from this study give varying, even conflicting, answers. The independent contrasts suggest that there is no mapping of function to geometry in inner curvature of pedal claws for digit III unguals. If independent contrasts are ignored, a relationship between behaviour and curvature appears evident, as it was in previous studies [2], [6], but we would like to emphasise that this is not straightforward, and that the multi-functionality of structures may well affect this relationship. Indeed, such relationships are expected due to the constraints placed on the analysis in behavioural category selection. There is still a need to better quantify an animal’s behaviour for future analyses. We might predict that species inhabiting habitats where small mistakes may lead to costly incidents exhibit some morphological specialisations. In addition, specialisations of other parts of the foot such as a reversed hallux, the modified digit II and raptorial claws of deinonychosaurs, or simply differentiation between the claw of digit III and other pedal claws, may confound these results.

Critically, however, our analyses (and indeed those of previous studies) do not take into account the relationship between the bony ungual and the keratinous claws. Preliminary results suggest that there is a significant difference between ungual-only and keratinous claw length (Binomial test; p<0.005) in extant taxa, with curvature decreasing and relative mid-point height increasing in ungual-only specimens. Despite the excellent soft tissue preservation of many small Mesozoic birds and other theropods, claw sheaths are rarely preserved; the data for some taxa are thus represented only by the bony ungual. When dealing with data from extant species, specimens in museums or other collections cannot easily be stripped of their keratin to allow examination of the underlying ungual. There is obviously some correlation between the shape of the ungual and the keratinous claw as a whole, but the relationship appears variable in extant taxa and few data are currently available. Consequently, extrapolations of ‘true’ claw shape in extinct animals based on ungual shape alone are problematic at best and research is required to better establish the relationship between ungual and keratin sheath size. Understanding the biomechanics of perching and climbing birds will allow better placement of fossil taxa in the context of our knowledge about extant animals, despite the acknowledged large overlap between the behavioural categories. It is also evident that better methods need to be determined for quantifying behaviour, in order that more robust analyses can be performed in future.

## Supporting Information

Table S1 **List of extant species used – asterisk in the source column denotes a specimen with only the ungual bone. ‘^Λ^’ denotes specimens measured following the same methodology detailed in the ‘Methods’ section (although printouts rather than ImageJ were used) but the data were taken from Miller (2005).**
(XLSX)

Table S2 **List of fossil specimen pedal claws used.**
(XLSX)

Table S3 **Citations for squamate masses.**
(DOCX)

Table S4 **Literature used to obtain phylogenies for separate bird orders.**
(DOCX)

Table S5 **Switched species list (see methods section for more details).**
(DOCX)

Table S6 **Species used from the Livezey & Zusi (2007) phylogeny for independent contrasts.**
(DOCX)

Table S7 **Species used from the Ericson et al., (2006) phylogeny for independent contrasts.**
(DOCX)

Table S8 **List of extant specimens used for the digit comparison analysis.**
(XLSX)
