# Supplementary material for: Pedal Claw Curvature in Birds, Lizards and Mesozoic Dinosaurs – Complicated Categories and Compensating for Mass-Specific and Phylogenetic Control
Source: PLoS One. 2012 Dec 5;7(12):e50555. doi: 10.1371/journal.pone.0050555 (PMC3515613; doi:10.1371/journal.pone.0050555)
Supplement: Table S3 — Citations for squamate masses. (DOCX) [file pone.0050555.s003.docx]

Supporting Information Table S3

Citations for squamate masses

| **Genus** | **Species** | **Reference** |
| --- | --- | --- |
| *Sphenodon* | *punctatus* | Herrell *et al.,* 2010 |
| *Agama* | *colonorum* | NaN |
| *Amblyrhynchus* | *cristatus* | French *et al.,* 2010 |
| *Chlamydosaurus* | *kingii* | Frappell & Mortola 1998 |
| *Cyclura* | *cornuta* | Fritz *et al.,* 2010 |
| *Iguana* | *sp.* | Fritz *et al.,* 2010 |
| *Lacerta* | *viridis* | Christian *et al.,* 1999 |
| *Polychrus* | *acutirostris* | NaN |
| *Tiliqua* | *scincoides* | Christian *et al.,* 2003 |
| *Tiliqua* | *sp.* | NaN |
| *Trachysaurus* | *rugosus* | Bull 1987 |
| *Uromastix* | *spinipes* | Castilla *et al.,* 2011 |
| *Varanus* | *albigularis* | Secor & Phillips 1997 |
| *Varanus* | *bengalensis* | Dryden *et al.,* 1992 |
| *Varanus* | *gouldii* | Frappell & Mortola 1998 |
| *Varanus* | *punctatus* | NaN |
| *Varanus* | *salvator* | Dryden *et al.,* 1992 |

References

**Bull CM. 1987.** A Population Study of the Viviparous Australian Lizard, Trachydosaurus rugosus (Scincidae). *Copeia* **1987:** 749-757.

**Castilla AM, Richer R, Herrel A, Conkey AAT, Tribuna J, Al-Thani M. 2011.** First evidence of scavenging behaviour in the herbivorous lizard Uromastyx aegyptia microlepis. *Journal of Arid Environments* **In Press, Corrected Proof**.

**Christian KA, Bedford GS, Schultz TJ. 1999.** Energetic consequences of metabolic depression in tropical and temperate-zone lizards. *Australian Journal of Zoology* **47:** 133-141.

**Christian KA, Webb JK, Schultz TJ. 2003.** Energetics of bluetongue lizards (*Tiliqua scincoides*) in a seasonal tropical environment. *Oecologia* **136:** 515-523.

**Dryden GL, Green B, Wikramanayake ED, Dryden KG. 1992.** Energy and Water Turnover in 2 Tropical Varanid Lizards, *Varanus bengalensis* and *Varanus salvator*. *Copeia***:** 102-107.

**Frappell PB, Mortola JP. 1998.** Passive body movement and gas exchange in the frilled lizard (Chlamydosaurus kingii) and goanna (Varanus gouldii). *Journal of Experimental Biology* **201:** 2307-2311.

**French SS, DeNardo DF, Greives TJ, Strand CR, Demas GE. 2010.** Human disturbance alters endocrine and immune responses in the Galapagos marine iguana (Amblyrhynchus cristatus). *Hormones and Behavior* **58:** 792-799.

**Fritz J, Hummel J, Kienzle E, Streich WJ, Clauss M. 2010.** To chew or not to chew: fecal particle size in herbivorous reptiles and mammals.  **313A:** 579-586.

**Herrel A, Moore JA, Bredeweg EM, Nelson NJ. 2010.** Sexual dimorphism, body size, bite force and male mating success in tuatara. *Biological Journal of the Linnean Society* **100:** 287-292.

**Secor SM, Phillips JA. 1997.** Specific Dynamic Action of a Large Carnivorous Lizard, *Varanus albigularis*. *Comparative Biochemistry and Physiology Part A: Physiology* **117:** 515-522.
