# Supplementary material for: Pedal Claw Curvature in Birds, Lizards and Mesozoic Dinosaurs – Complicated Categories and Compensating for Mass-Specific and Phylogenetic Control
Source: PLoS One. 2012 Dec 5;7(12):e50555. doi: 10.1371/journal.pone.0050555 (PMC3515613; doi:10.1371/journal.pone.0050555)
Supplement: Table S4 — Literature used to obtain phylogenies for separate bird orders. (DOCX) [file pone.0050555.s004.docx]

Supporting Information Table S4

Literature used to obtain phylogenies for separate bird orders

| **Order** | **Literature** |
| --- | --- |
| Galliformes | Crowe *et al.,* 2006 |
| Procellariiformes | Penhallurick & Wink 2004 |
| Ciconiiformes | McCracken & Sheldon 1998 |
| Gruiformes | Fain *et al.,* 2007 |
| Charadriiformes | Thomas *et al.,* 2004 |
| Falconiformes | Sibley & Ahlquist 1990 |
| Cuculiformes | Aragon *et al.,* 1999 |
| Columbiformes | Johnson & Clayton 2000 |
| Piciformes | Webb & Moore 2005 |
| Passeriformes - Tyranni | Chesser 2004 |
| Passeriformes - Passeri | [Jønsson & Fjeldså](#_ENREF_15) 2006 |

References

**Aragon S, Moller AP, Soler JJ, Soler M. 1999.** Molecular phylogeny of cuckoos supports a polyphyletic origin of brood parasitism. *Journal of Evolutionary Biology* **12:** 495-506.

**Chesser RT. 2004.** Molecular systematics of New World suboscine birds. *Molecular Phylogenetics and Evolution* **32:** 11-24.

**Crowe TM, Bowie RCK, Bloomer P, Mandiwana TG, Hedderson TAJ, Randi E, Pereira SL, Wakeling J. 2006.** Phylogenetics, biogeography and classification of, and character evolution in, gamebirds (Aves : Galliformes): effects of character exclusion, data partitioning and missing data. *Cladistics* **22:** 495-532.

**Fain MG, Krajewski C, Houde P. 2007.** Phylogeny of "core Gruiformes" (Aves : Grues) and resolution of the Limpkin-Sungrebe problem. *Molecular Phylogenetics and Evolution* **43:** 515-529.

**Johnson KP, Clayton DH. 2000.** Nuclear and mitochondrial genes contain similar phylogenetic signal for pigeons and doves (Aves : Columbiformes). *Molecular Phylogenetics and Evolution* **14:** 141-151.

**Jønsson KA, Fjeldså J. 2006.** A phylogenetic supertree of oscine passerine birds (Aves: Passeri). *Zoologica Scripta* **35:** 149-186.

**McCracken KG, Sheldon FH. 1998.** Molecular and osteological heron phylogenies: Sources of incongruence. *Auk* **115:** 127-141.

**Penhallurick J, Wink M. 2004.** Analysis of the taxonomy and nomenclature of the Procellariiformes based on complete nucleotide sequences of the mitochondrial cytochrome b gene. *Emu* **104:** 125-147.

**Sibley CG, Ahlquist JE. 1990.** *Phylogeny and classification of birds : a study in molecular evolution*. Yale University Press: New Haven.

**Thomas GH, Wills MA, Szekely T. 2004.** Phylogeny of shorebirds, gulls, and alcids (Aves : Charadrii) from the cytochrome-b gene: parsimony, Bayesian inference, minimum evolution, and quartet puzzling. *Molecular Phylogenetics and Evolution* **30:** 516-526.

**Webb DM, Moore WS. 2005.** A phylogenetic analysis of woodpeckers and their allies using 12S, Cyt b, and COI nucleotide sequences (class Aves; order Piciformes). *Molecular Phylogenetics and Evolution* **36:** 233-248.
