# Supplementary material for: Pedal Claw Curvature in Birds, Lizards and Mesozoic Dinosaurs – Complicated Categories and Compensating for Mass-Specific and Phylogenetic Control
Source: PLoS One. 2012 Dec 5;7(12):e50555. doi: 10.1371/journal.pone.0050555 (PMC3515613; doi:10.1371/journal.pone.0050555)
Supplement: Table S5 — Switched species list (see methods section for more details). (DOCX) [file pone.0050555.s005.docx]

Supporting Information Table S5

Switched species list (see methods section for more details)

| **Genus** | **Species from data collection** | **Species from phylogeny in literature** | **Order** |
| --- | --- | --- | --- |
| *Ardea* | *cinerea* | *alba* | Ciconiiformes |
| *Botaurus* | *stellaris* | *lentiginosus* | Ciconiiformes |
| *Ciconia* | *ciconia* | *alba* | Ciconiiformes |
| *Ixobrychus* | *flavicollis* | *exillis* | Ciconiiformes |
| *Charadrius* | *hiaticula* | *alexandrinus* | Charadriiformes |
| *Larus* | *canus* | *novaehollandiae* | Charadriiformes |
| *Stercorarius* | *parasiticus* | *pomarinus* | Charadriiformes |
| *Tringa* | *nebularia* | *glareola* | Charadriiformes |
| *Accipiter* | *gentilis* | *bicolor* | Falconiformes |
| *Accipiter* | *nisus* | *cooperii* | Falconiformes |
| *Elanus* | *caeraleus* | *leucurus* | Falconiformes |
| *Columba* | *palumbus* | *leucocephala* | Columbiformes |
| *Ptinilopus* | *melanospila* | *leclancheri* | Columbiformes |
| *Streptopelia* | *turtur* | *chinensis* | Columbiformes |
| *Megalaima* | *mystacophanos* | *haemacephala* | Piciformes |
| *Picoides* | *arcticus* | *albolarvatus* | Piciformes |
| *Picoides* | *major* | *pubescens* | Piciformes |
| *Lichmera* | *lombokia* | *indistincta* | Passeriformes |
| *Cracticus* | *nigrogularis* | *quoyi* | Passeriformes |
| *Dicrurus* | *macrocercus* | *adsimilis* | Passeriformes |
| *Troglodytes* | *troglodytes* | *aedon* | Passeriformes |
| *Mycerobas* | *icteroides* | *carnipes* | Passeriformes |
| *Ploceus* | *velatus* | *ocularis* | Passeriformes |
| *Eremophila* | *alpetris* | *bilopha* | Passeriformes |
| *Psaltriparus* | *melanotus* | *minimus* | Passeriformes |
| *Pycnonotus* | *capensis* | *atriceps* | Passeriformes |
