# Supplementary material for: Pedal Claw Curvature in Birds, Lizards and Mesozoic Dinosaurs – Complicated Categories and Compensating for Mass-Specific and Phylogenetic Control
Source: PLoS One. 2012 Dec 5;7(12):e50555. doi: 10.1371/journal.pone.0050555 (PMC3515613; doi:10.1371/journal.pone.0050555)
Supplement: Table S6 — Species used from the Livezey & Zusi (2007) phylogeny for independent contrasts. (DOCX) [file pone.0050555.s006.docx]

Supporting Information Table S6

Species used from the Livezey & Zusi (2007) phylogeny for independent contrasts

| **Genus** | **Order** |
| --- | --- |
| *Apteryx* | Apterygiformes |
| *Ardea* | Ardeiformes |
| *Nycticorax* | Ardeiformes |
| *Botaurus* | Ardeiformes |
| *Balaeniceps* | Balaenicipitiformes |
| *Steatornis* | Caprimulgiformes |
| *Podargus* | Caprimulgiformes |
| *Rostratula* | Charadriiformes |
| *Pluvialis* | Charadriiformes |
| *Glareola* | Charadriiformes |
| *Burhinus* | Charadriiformes |
| *Haematopus* | Charadriiformes |
| *Himantopus* | Charadriiformes |
| *Chionis* | Charadriiformes |
| *Stercorarius* | Charadriiformes |
| *Rissa* | Charadriiformes |
| *Plegadis* | Ciconiiformes |
| *Ciconia* | Ciconiiformes |
| *Colius* | Coliiformes |
| *Pterocles* | Columbiformes |
| *Columba* | Columbiformes |
| *Merops* | Coraciiformes |
| *Leptosomus* | Coraciiformes |
| *Phoeniculus* | Coraciiformes |
| *Tockus* | Coraciiformes |
| *Momotus* | Coraciiformes |
| *Alcedo* | Coraciiformes |
| *Geococcyx* | Cuculiformes |
| *Centropus* | Cuculiformes |
| *Cuculus* | Cuculiformes |
| *Cathartes* | Falconiformes |
|  |  |
| **Genus** | **Order** |
| *Sagittarius* | Falconiformes |
| *Accipiter* | Falconiformes |
| *Gyps* | Falconiformes |
| *Pandion* | Falconiformes |
| *Falco* | Falconiformes |
| *Megapodius* | Galliformes |
| *Ortalis* | Galliformes |
| *Meleagris* | Galliformes |
| *Alectoris* | Galliformes |
| *Numida* | Galliformes |
| *Psophia* | Gruiformes |
| *Grus* | Gruiformes |
| *Pitta* | Passeriformes |
| *Pitangus* | Passeriformes |
| *Ptilonorhynchus* | Passeriformes |
| *Bombycilla* | Passeriformes |
| *Parus* | Passeriformes |
| *Passer* | Passeriformes |
| *Galbula (Urogalba)* | Piciformes |
| *Megalaima* | Piciformes |
| *Picoides* | Piciformes |
| *Jynx* | Piciformes |
| *Pelecanoides* | Procellariiformes |
| *Puffinus* | Procellariiformes |
| *Diomedea* | Procellariiformes |
| *Trichoglossus* | Psittaciformes |
| *Cacatua* | Psittaciformes |
| *Tyto* | Strigiformes |
| *Phodilus* | Strigiformes |
| *Strix* | Strigiformes |
| *Otus* | Strigiformes |

References

**Livezey BC, Zusi RL. 2007.** Higher-order phylogeny of modern birds (Theropoda, Aves : Neornithes) based on comparative anatomy. II. Analysis and discussion. *Zoological Journal of the Linnean Society* **149:** 1-95.
