# Supplementary material for: Pedal Claw Curvature in Birds, Lizards and Mesozoic Dinosaurs – Complicated Categories and Compensating for Mass-Specific and Phylogenetic Control
Source: PLoS One. 2012 Dec 5;7(12):e50555. doi: 10.1371/journal.pone.0050555 (PMC3515613; doi:10.1371/journal.pone.0050555)
Supplement: Table S7 — Species used from the Ericson et al., (2006) phylogeny for independent contrasts. (DOCX) [file pone.0050555.s007.docx]

Supporting Information Table S7

Species used from the Ericson *et al.,* (2006) phylogeny for independent contrasts

| **Genus** | **Family** |
| --- | --- |
| *Accipiter* | Accipitridae |
| *Alcedo* | Alcedinidae |
| *Apteryx* | Apterygidae |
| *Ardea* | Ardeidae |
| *Balaeniceps* | Balaenicipitidae |
| *Tockus* | Bucerotidae |
| *Cacatua* | Cacatuidae |
| *Megalaima* | Capitonidae |
| *Coragyps* | Cathartidae |
| *Cathartes* | Cathartidae |
| *Charadrius* | Charadriidae |
| *Colius* | Coliidae |
| *Cuculus* | Cuculidae |
| *Diomedea* | Diomedeidae |
| *Falco* | Falconidae |
| *Galbula (Urogalba)* | Galbulidae |
| *Grus* | Gruidae |
| *Lanius* | Laniidae |
| **Genus** | **Family** |
| *Leptosomus* | Leptosomidae |
| *Alectura* | Megapodiidae |
| *Merops* | Meropidae |
| *Momotus* | Momotidae |
| *Opisthocomus* | Opisthocomidae |
| *Pandion* | Pandionidae |
| *Pelecanoides* | Pelecanoididae |
| *Podargus* | Podargidae |
| *Puffinus* | Procellariidae |
| *Fulmarus* | Procellariidae |
| *Psophia* | Psophiidae |
| *Pterocles* | Pteroclididae |
| *Rhea* | Rheidae |
| *Sagittarius* | Sagittariidae |
| *Steatornis* | Steatornithidae |
| *Asio* | Strigidae |
| *Turnix* | Turnicidae |
| *Tyto* | Tytonidae |

References

**Ericson PGP, Anderson CL, Britton T, Elzanowski A, Johansson US, Kallersjo M, Ohlson JI, Parsons TJ, Zuccon D, Mayr G. 2006.** Diversification of Neoaves: integration of molecular sequence data and fossils. *Biology Letters* **2:** 543-547.
